# Repeated Detection of Vaccine-Preventable Anal HPV Genotypes in a Screened Cohort: A Retrospective Observational Study

**DOI:** 10.3390/pathogens15070730

**Published:** 2026-07-10

**Authors:** Alberto Rizzo, Davide Moschese, Federica Salari, Luca Carsana, Samuel Lazzarin, Andrea Cavallo, Chiara Fusetti, Loriana Morelli, Francesco Caruso, Alessandra Lombardi, Andrea Giacomelli, Manuela Nebuloni, Alberto Dolci, Andrea Gori

**Affiliations:** 1 Laboratory of Clinical Microbiology, Virology and Bioemergencies, Luigi Sacco Hospital, Regional Center for Infectious Diseases (CEREMI), Lombardy Region, 20157 Milan, Italy; salari.federica@asst-fbf-sacco.it (F.S.); cavallo.andrea@asst-fbf-sacco.it (A.C.); loriana.morelli@asst-fbf-sacco.it (L.M.); lombardi.alessandra@asst-fbf-sacco.it (A.L.); alberto.dolci@unimi.it (A.D.); 2 Department of Infectious Disease, Luigi Sacco Hospital, Regional Center for Infectious Diseases (CEREMI), Lombardy Region, 20157 Milan, Italy; davide.moschese@unimi.it (D.M.); lazzarin.samuel@asst-fbf-sacco.it (S.L.); fusetti.chiara@asst-fbf-sacco.it (C.F.); caruso.francesco@asst-fbf-sacco.it (F.C.); andrea.giacomelli@unimi.it (A.G.); andrea.gori@unimi.it (A.G.); 3 Department of Biomedical and Clinical Sciences, Università degli Studi di Milano, 20157 Milan, Italy; manuela.nebuloni@unimi.it; 4 Pathology Unit, Luigi Sacco Hospital, 20157 Milan, Italy; carsana.luca@asst-fbf-sacco.it

**Keywords:** HPV, HPV16, repeated detection, cytology, PLWH, MSM

## Abstract

The clinical relevance of concurrent vaccine-preventable anal HPV genotype infections, particularly for short-term repeated detection and cytologic outcomes in screened high-risk populations, remains uncertain. We performed a single-centre retrospective study of 145 adults with at least one 9-valent vaccine-preventable anal HPV genotype at baseline and repeat HPV DNA testing and cytology approximately 12 months later. Baseline infections were classified as single- or multiple-genotype infections. Outcomes included clearance of all baseline vaccine-preventable genotypes, genotype-specific repeated detection, detection of new vaccine-preventable genotypes, complete HPV negativity, and cytologic regression or progression. At baseline, 70 participants (48.3%) had single and 75 (51.7%) had multiple genotype infections; most were male (95.2%) and people living with HIV (88.3%), reflecting a highly selected anal HPV-screened cohort. Multiple infections were associated with more frequent abnormal baseline cytology (69.3% vs. 45.7%). At follow-up, clearance of all baseline vaccine-preventable genotypes was more common after single than multiple infections (34.3% vs. 12.0%). Complete HPV negativity was also more frequent after single infection (24.3% vs. 4.0%), although this stringent endpoint is intrinsically more difficult to achieve in individuals with multiple baseline infections. New vaccine-preventable genotype detection did not differ significantly between groups. Genotype-specific repeated detection was generally similar between groups, except for HPV58, a finding based on small subgroup numbers. Cytologic regression was more frequent after single infection, but not statistically significant. Multiple vaccine-preventable anal HPV genotype infections were associated with greater baseline cytologic abnormality and lower clearance of baseline vaccine-preventable genotypes at 12 months. Given the retrospective design, selected population, limited event counts, and residual confounding, these findings should be interpreted as hypothesis-generating and do not prove impaired biological clearance.

## 1. Introduction

Human Papillomavirus (HPV), one of the most common sexually transmitted infections (STIs), is a major cause of anal intraepithelial neoplasia and invasive cancer [1,2,3]. High-risk HPV genotypes, particularly those included in the nonavalent vaccine (9vHPV), account for the majority of anal lesion cases worldwide. The concurrent presence of multiple HPV genotypes is common, particularly among people living with HIV (PLWH) and men who have sex with men (MSM), populations in whom anal HPV prevalence and persistence are substantially higher than in the general population [4,5,6]. However, the clinical significance of multiple infections on viral persistence and HPV-associated abnormalities remains unclear [7,8]. Most prior studies have focused on the presence of high-risk HPV overall rather than distinguishing between single and multiple genotype anal infections, and longitudinal data evaluating type-specific persistence in screened clinical populations are limited [9,10]. While concurrent infection with multiple genotypes may reflect increased exposure risk or host susceptibility, it is uncertain whether multiple infections are independently associated with differences in viral clearance or cytological outcomes, or whether observed associations are driven by behavioral or immunologic confounding. In addition, vaccine-preventable genotypes represent a clinically relevant subgroup given their contribution to anal cancer burden and the potential implications for prevention strategies. Understanding the natural history of these genotypes in individuals undergoing anal screening may help contextualize follow-up findings without implying causal mechanisms. This study aimed to evaluate the 12-month repeated detection and clearance of vaccine-preventable anal HPV types in a screened cohort and to compare individuals with single versus multiple HPV genotype infections at baseline. Specifically, we examined genotype-specific repeated detection, clearance of baseline vaccine-preventable genotypes, new genotype detection, complete HPV negativity, and changes in anal cytology. Exploratory analyses according to HPV vaccination status were also performed.

## 2. Materials and Methods

### 2.1. Study Design

This single-centre, retrospective observational study included patients undergoing anal HPV DNA testing and concurrent anal cytology at the Infectious Diseases Department, ASST Fatebenefratelli Sacco Hospital, Milan, from 2022 to 2023. The study included adult individuals (aged ≥ 18 years) who tested positive for at least one vaccine-preventable HPV genotype at baseline HPV DNA testing (T0) and who had a follow-up visit (T1) with repeated anal HPV testing and cytology at approximately 12 months. The median interval between T0 and T1 was 13 (interquartile range: 11–16) months. Because this was a retrospective routine-care cohort, follow-up timing was not fixed by protocol. The T1 visit corresponded to the available repeat anal HPV DNA test and cytology performed approximately one year after T0 as part of clinical care. Analyses were conducted using available data for each endpoint. Variables collected included age, assigned sex at birth, HPV vaccination and HIV status at baseline. For PLWH, HIV viral load values obtained within ±1 month of HPV testing at T0 and T1 were collected when available. Information on CD4 count, smoking status, detailed sexual behavior characteristics, previous HPV-associated disease, and detailed prior anal history were not consistently collected in the retrospective dataset.

Anal samples were collected using a cytobrush and ThinPrep™ (Hologic BV, Zaventem, Belgium) for the HPV DNA testing, while for the cytological examination two direct smear slides were prepared for each patient. Anal HPV testing was performed as part of routine screening in individuals attending the infectious diseases clinic, mainly because of reported unprotected anal intercourse with at least one partner in the previous 3 months and/or a history of other STIs, to rule out potential causes of anal inflammation and abnormalities.

### 2.2. Laboratory Testing

HPV DNA detection, including vaccine-preventable types (HPV16, HPV18, HPV31, HPV33, HPV45, HPV52, HPV58, HPV6, and HPV11), was performed at both time points using the real-time PCR Allplex™ HPV28 Detection (Seegene Inc., Seoul, Republic of Korea) commercial assay. Infections were categorized as single infection (only one 9vHPV-preventable HPV genotype detected) or multiple infection (more than one 9vHPV-preventable genotype detected).

Complete HPV negativity at T1 was operationally defined as the absence of all HPV genotypes evaluated in the present analysis at follow-up. This endpoint was selected as a conservative and clinically intuitive measure of complete detectable HPV resolution. It differs from genotype-specific clearance, because participants were counted as completely HPV-negative only if no evaluated genotype was detected at T1, including baseline genotypes and any newly detected genotypes. Therefore, complete HPV negativity is intrinsically more stringent for participants with multiple baseline infections, because all evaluated genotypes must be absent at follow-up. Clearance of all baseline vaccine-preventable genotypes was defined as absence at T1 of every vaccine-preventable genotype detected at T0, irrespective of newly detected vaccine-preventable genotypes at T1. New vaccine-preventable genotype detection was defined as detection at T1 of at least one vaccine-preventable genotype not detected at T0. Genotype-specific repeated detection was defined as detection at T1 of the same HPV genotype present at T0. Because HPV testing was performed at only two time points, this endpoint was not considered definitive proof of continuous infection and may reflect continuous infection, intermittent detectability, or reinfection with the same genotype. Genotype-specific clearance was defined as absence at T1 of a genotype detected at T0, irrespective of other HPV genotypes detected at follow-up. Anal cytology was reported according to the 2014 Bethesda System for Reporting Cervical Cytology [11]. Baseline anal cytology was classified as NEG (unremarkable), LSIL (low-grade squamous intraepithelial lesion), ASC (atypical squamous cells), or HSIL (high-grade squamous intraepithelial lesion). Cytology regression was defined as transition from an abnormal cytology result (ASC, LSIL, or HSIL) at T0 to a negative (NEG) result at T1, whereas cytology progression was defined as a change from NEG at T0 to an abnormal result (ASC, LSIL, or HSIL) at T1.

### 2.3. Statistics

Descriptive statistics were used to summarize demographic and clinical characteristics of the study population. Continuous variables were presented as median and interquartile range (IQR), while categorical variables were presented as counts and percentages. Categorical variables were compared using Chi-square test or Fisher’s exact test when expected cell counts were small. Continuous variables were compared using the Mann–Whitney U test.

An exploratory multivariable logistic regression model was fitted only for abnormal baseline cytology (ASC, LSIL, or HSIL) versus negative cytology, because this was the outcome with the most robust event count. The exposure of interest was baseline infection pattern, defined as multiple versus single vaccine-preventable HPV genotype infection. The model was adjusted for age, HPV vaccination status, and HIV status. Adjusted odds ratios (ORs) with 95% confidence intervals (CIs) were reported. Multivariable models were not fitted for complete HPV negativity at T1, clearance of all T0 vaccine-preventable genotypes, cytologic regression, cytologic progression, or genotype-specific repeated detection because of limited event numbers, small genotype-specific denominators, and the risk of overfitting. Variables not systematically collected, including detailed sexual behavior, smoking status, CD4 count, prior STI history, previous HPV-associated disease, and previous anal screening history, were not included in the statistical models.

A *p*-value < 0.05 was considered statistically significant.

## 3. Results

### 3.1. Characteristics of the Population

Overall, the study included 145 participants. Of these, 70 (48.3%) had a single HPV genotype infection at T0, while 75 (51.7%) had multiple genotype infections. The cohort was predominantly male (138/145, 95.2%), with a median age of 47 (38–54) years and 128 PLWH participants (88.3%), indicating a selected anal HPV-screened cohort. Among PLWH, HIV viral load data were available for all 128 participants. At T0, 127/128 (99.2%) had HIV RNA < 200 copies/mL, while one participant had HIV RNA ≥ 200 copies/mL. At T1, all PLWH had HIV RNA < 200 copies/mL.

At T0, 38 of 145 (26.2%) participants had received at least one dose of nonavalent HPV vaccine. Specifically, 34 individuals completed the three-dose vaccination schedule before T0 testing (median time from vaccination to testing: 0.8 [0.2–2.3] years). Regarding cytology, 61/145 (42.1%) participants had negative cytology (NEG) and 84/145 (57.9%) had abnormalities, defined as ASC, LSIL, or HSIL.

### 3.2. Characteristics of Individuals with Single vs. Multiple Genotype Infections

In the single infection group (*n* = 70), 65 (92.9%) individuals were male, the median age was 46 (37–53) years, 62 (88.6%) were PLWH, 21 (30.0%) were vaccinated, and 32 (45.7%) had abnormal cytology at T0. In contrast, in the multiple infection group (*n* = 75), 73 (97.3%) individuals were male, the median age was 48 (37–56) years, 66 (88.0%) were PLWH, 17 (22.7%) were vaccinated, and 52 (69.3%) had abnormal cytology at T0. Table 1 shows the characteristics of individuals with single and multiple HPV genotype infections.

After adjustment for age, HPV vaccination status, and HIV status, multiple baseline vaccine-preventable HPV genotype infection remained associated with higher odds of abnormal T0 cytology compared with single infection (adjusted OR, 2.65; 95% CI, 1.33–5.28; *p* = 0.006; Appendix A, Table A1). This analysis was exploratory, and the primary analyses remained the univariate comparisons.

The T0 distribution of specific HPV genotypes differed between groups. In the single infection group (*n* = 70), HPV16 was detected in 19 cases (27.1%), HPV58 in 12 cases (17.1%), and HPV6 in 10 cases (14.3%). In the multiple infection group (*n* = 75), HPV16 was the most prevalent genotype (39, 52.0%), followed by HPV58 (35, 46.7%) and HPV52 (17/75, 22.7%). Table 2 shows the distribution of HPV genotypes in the single infection and in the multiple infection groups.

Overall, complete HPV negativity at T1 was achieved in 20/145 (13.8%) individuals. Specifically, it was observed in 17/70 individuals (24.3%) with single infection and 3/75 individuals (4.0%) with multiple infections (*p* < 0.001). Because complete HPV negativity required absence of all evaluated genotypes at T1, this result is intrinsically more difficult to achieve in participants with multiple baseline infections and should not be interpreted as a direct measure of genotype-specific biological clearance.

When considering clearance of vaccine-preventable genotypes irrespective of the presence of newly detected HPV types at T1, a higher proportion of individuals in the single infection group cleared all T0 genotypes compared with those with multiple infections (24/70 [34.3%] vs. 9/75 [12.0%], *p* = 0.002). New vaccine-preventable HPV genotypes at T1 were detected in 39/145 (26.9%) cases; specifically, in 16/70 (22.9%) individuals with single infection and in 23/75 (30.7%) individuals with multiple infections at T0 (*p* = 0.350). This finding indicates that new vaccine-preventable genotype detection during follow-up was common in this screened high-risk population and may have contributed to the low proportion of participants reaching the complete HPV-negativity endpoint.

Most genotype-specific repeated detection rates were similar between groups. Repeated HPV58 detection differed between groups (12/12, 100%, in the single-infection group vs. 22/35, 62.9%, in the multiple-infection group; *p* = 0.021), although this finding was based on a small subgroup, particularly in the single-infection group, and should be considered exploratory (Table 3).

Among participants with abnormal T0 cytology, cytologic regression to NEG at T1 was observed in 17/32 (53.1%) individuals in the single-infection group and 16/52 (30.8%) in the multiple-infection group (*p* = 0.065). Among participants with NEG cytology at T0, cytologic progression to ASC, LSIL, or HSIL at T1 occurred in 9/38 (23.7%) single-infection cases and 8/23 (34.8%) multiple-infection cases (*p* = 0.388). These cytologic outcomes were based on cytology only and were not systematically confirmed by high-resolution anoscopy or histology.

### 3.3. Baseline Characteristics and 12-Month Outcomes According to HPV Vaccination Status

Analyses according to HPV vaccination status were exploratory and were not designed to estimate vaccine effectiveness. At baseline, 38/145 individuals (26.2%) had received at least one dose of HPV vaccine. Vaccinated individuals were significantly younger than unvaccinated individuals (median age 40 [IQR 35–47] years vs. 50 [IQR 39–56] years, *p* < 0.001), representing an important potential confounder for vaccination-stratified comparisons. The distribution of assigned sex at birth and HIV status was similar between groups. Regarding T0 cytology, vaccinated individuals showed a higher proportion of normal cytology (21/38, 55.3%) compared with unvaccinated individuals (40/107, 37.4%), although this difference did not reach statistical significance (*p* = 0.055). LSIL was less frequent among vaccinated individuals (5/38, 13.1%) than among unvaccinated individuals (42/107, 39.2%; *p* = 0.004). Table 4 summarizes baseline characteristics according to vaccination status.

Most genotype-specific detection rates at T0 were similar between groups; however, HPV16 was more frequent among unvaccinated individuals (*p* = 0.045). The distribution of other vaccine-preventable HPV genotypes did not differ significantly between groups (Table 5).

At T1, complete HPV negativity was observed in 8/38 (21.0%) vaccinated individuals and 12/107 (11.2%) unvaccinated individuals (*p* = 0.131). Cytologic regression occurred in 10/17 (58.8%) vaccinated individuals compared with 23/67 (34.3%) unvaccinated individuals (*p* = 0.065), while cytologic progression was observed in 8/21 (38.1%) vaccinated individuals and 9/40 (22.5%) unvaccinated individuals (*p* = 0.197).

## 4. Discussion

In this retrospective cohort of predominantly male individuals (95.2%), most of whom were PLWH (88.3%) with near-universal virological suppression, multiple vaccine-preventable anal HPV genotype infections at baseline were associated with a higher prevalence of cytologic abnormalities and lower clearance of all baseline vaccine-preventable genotypes at 12 months compared with single genotype infections. This is consistent with previous evidence showing that anal HPV infection and HPV-associated abnormalities are highly prevalent among MSM and PLWH [12]. These findings add longitudinal data to the limited literature comparing single and multiple anal HPV genotype infections in screened clinical populations, but they should be interpreted in the context of a selected high-risk population attending an infectious diseases setting.

The higher prevalence of abnormal cytology observed among individuals with multiple infections at T0 may reflect several non-mutually exclusive factors. Multiple genotype detection may represent increased cumulative HPV exposure, shared behavioral risk factors, host susceptibility, or immunological vulnerability, particularly in a cohort with a high prevalence of HIV. Alternatively, a higher total genotype burden may increase the probability of detecting cytologic abnormalities without necessarily implying synergistic biological interaction between viral types. Therefore, in this study, multiplicity should be interpreted primarily as a marker of exposure burden or clinical risk rather than as evidence that multiple infections directly impair genotype-specific clearance. Similar associations between multiple HPV-type detection and anal precancerous lesions have been reported in men living with HIV, supporting the interpretation that genotype burden may be a marker of lesion risk rather than evidence of direct viral synergy [13]. Studies of MSM undergoing anal cancer screening have similarly shown that multiple HPV infections are frequent and often coexist with cytologic or histologic abnormalities, reinforcing the need to interpret multiplicity as a clinical-risk marker [14]. Multiple HPV genotype detection may also be a marker of broader sexual network exposure and cumulative STI or pathogen burden. In high-risk screened populations, repeated exposure to diverse pathogens may contribute to mucosal inflammation or immune activation and could influence HPV detection and cytologic abnormalities. However, the present dataset did not include detailed partner number, contemporaneous STI diagnoses, or markers of other viral coinfections; therefore, this hypothesis could not be directly evaluated.

When clearance of all T0 vaccine-preventable genotypes was examined, a higher proportion was observed in the single-infection group (34.3% vs. 12.0%). This endpoint is more directly interpretable than complete HPV negativity because it focuses on baseline vaccine-preventable genotypes, irrespective of newly detected types at follow-up. Complete HPV negativity was retained only as a stringent secondary endpoint and should not be interpreted alone as evidence of impaired biological clearance, because it required absence of all evaluated genotypes at T1 and was therefore intrinsically more difficult to achieve for individuals with multiple baseline infections. For this reason, the interpretation of follow-up HPV outcomes was based primarily on clearance of baseline vaccine-preventable genotypes, genotype-specific repeated detection, and new genotype detection.

The detection of new vaccine-preventable HPV genotypes at follow-up in approximately 27% of participants highlights the dynamic nature of anal HPV detection in this population. New genotype detection may reflect ongoing sexual exposure, reinfection, reactivation of previously undetectable infection, intermittent detectability, or susceptibility related to host or immunological factors. Although new genotype detection did not differ significantly between groups, it may have contributed to the low frequency of complete HPV negativity, particularly among individuals with multiple baseline infections. These findings support interpreting 12-month anal HPV outcomes as a balance between clearance of baseline genotypes, repeated detection of baseline genotypes, and detection of new genotypes rather than as a simple persistence-versus-clearance dichotomy. Incident anal HPV detection and type-specific persistence have been described as common events in longitudinal studies of men, particularly among MSM, supporting the interpretation that new genotype detection during follow-up may reflect ongoing exposure [15]. In PLWH as well, anal HPV detection reflects a dynamic balance between incidence, persistence, and clearance, further supporting the need to distinguish complete HPV negativity from genotype-specific clearance [16].

Repeated HPV58 detection was numerically higher in the single-infection group. However, this result was based on only 12 HPV58-positive participants in the single-infection group and should be considered exploratory. No inference regarding HPV58-specific viral fitness, transmissibility, or host immune control can be made from this subgroup finding.

Taken together, the lower likelihood of complete HPV negativity and lower clearance of all T0 vaccine-preventable genotypes among individuals with multiple infections may reflect baseline genotype burden, repeated detection of at least one baseline genotype, and newly detected HPV types during follow-up rather than a uniform impairment in genotype-specific clearance. This interpretation is consistent with the dynamic natural history of HPV infection, which may include persistence, clearance, intermittent detection, reactivation, and reinfection, and with longitudinal data showing that high-risk anal HPV persistence in MSM is influenced by sexual exposure patterns [17,18].

Cytologic regression, assessed as a screening-test outcome rather than a histologically confirmed lesion outcome, appeared more frequent in the single-infection group, although subgroup analyses were limited by small numbers. These findings suggest that infection burden may be associated with short-term cytologic dynamics; however, cytology outcomes were not systematically confirmed by histology, and misclassification cannot be excluded [19]. Residual confounding, particularly related to sexual behavior, smoking, HIV-related immunologic markers, and prior screening history, may influence both infection multiplicity and cytologic evolution. Although HIV viral load was available for all PLWH and showed near-universal virological suppression at both time points, residual HIV-related confounding cannot be excluded because CD4 count, nadir CD4 count, duration of HIV infection, and ART history were not consistently available.

In exploratory analyses stratified by HPV vaccination status, vaccinated individuals were significantly younger than unvaccinated individuals. This age imbalance is a major potential confounder, and the present retrospective study was not designed to assess HPV vaccine effectiveness. Although vaccinated participants showed a numerically higher frequency of complete HPV negativity and cytologic regression, these differences were not statistically significant in univariate analyses and should be considered hypothesis-generating only. The lower baseline prevalence of HPV16 among vaccinated participants should likewise be interpreted cautiously because vaccination status was not randomized and may be associated with age, sexual exposure, screening history, and other unmeasured factors. In addition, exact timing of vaccination relative to HPV acquisition and complete dose-level information were not available for all participants. The focus on vaccine-preventable genotypes was chosen because these types are included in the nonavalent HPV vaccine and are relevant to prevention strategies; however, the present study was not designed to evaluate prophylactic or therapeutic vaccine effectiveness. Stronger conclusions regarding vaccine effectiveness would require prospective studies with detailed vaccination histories and appropriate adjustment for confounding.

This study has several limitations. First, the cohort was highly selected, consisting predominantly of male participants and PLWH undergoing anal HPV screening in an infectious diseases setting; therefore, the findings may not be generalizable to women, HIV-negative individuals, unscreened populations, or general population screening cohorts. Second, the modest sample size and limited number of events for several endpoints reduced statistical power, particularly for complete HPV negativity at T1, clearance of all baseline vaccine-preventable genotypes, cytologic regression, cytologic progression, and genotype-specific repeated detection. Accordingly, only one exploratory multivariable logistic regression model was fitted, for abnormal baseline cytology, while additional multivariable models were not performed to avoid overfitting and unstable estimates. Third, residual confounding remains likely because key behavioral, clinical, and immunological variables, including detailed sexual exposure, smoking status, prior STI history, previous HPV-associated disease, previous anal screening history, CD4 count, and HPV viral load, were not available or not consistently captured. Fourth, vaccination-stratified analyses were limited by incomplete information and cannot be interpreted as estimates of vaccine effectiveness. Fifth, cytologic outcomes were not systematically confirmed by high-resolution anoscopy-guided biopsy or histology. Cytology-only assessment can misclassify lesion status and may either overestimate or underestimate true histologic regression or progression, particularly for clinically relevant anal HSIL. Therefore, cytologic regression and progression should be interpreted as screening-test dynamics rather than confirmed lesion outcomes. Sixth, HPV repeated detection was assessed using DNA detection at only two time points. Detection of the same genotype at T0 and T1 was classified analytically as genotype-specific repeated detection rather than confirmed persistence, because this approach cannot distinguish continuous infection from intermittent detectability, viral latency with reactivation, or reinfection with the same genotype. Viral sequencing and more frequent longitudinal sampling would be required to distinguish these mechanisms. Finally, analyses focused on vaccine-preventable genotypes, and conclusions should not be extended to non-vaccine-preventable HPV types.

Despite these limitations, this study provides real-world longitudinal data on vaccine-preventable anal HPV genotypes in a high-risk screened population. By distinguishing single from multiple genotype infections, it highlights short-term virologic and cytologic patterns that merit further investigation. Larger prospective cohorts incorporating more frequent sampling, viral sequencing, quantitative HPV viral load, HIV-related immunological markers, detailed behavioral and vaccination data, histologic confirmation, and non-vaccine-preventable HPV genotypes are needed to better characterize the clinical significance of concurrent vaccine-preventable HPV infections.

## 5. Conclusions

In summary, in this highly selective, screened cohort composed predominantly of male PLWH, multiple vaccine-preventable HPV genotype infections were associated with a higher prevalence of anal cytologic abnormalities and lower clearance of all baseline vaccine-preventable genotypes at 12 months. Repeated detection of baseline genotypes and detection of new vaccine-preventable genotypes were common, highlighting the dynamic nature of anal HPV detection in this population. Given the retrospective design, limited event counts, residual confounding, cytology-only outcome assessment, and the availability of only two HPV testing time points, these findings may reflect differences in exposure burden, host susceptibility, intermittent detectability, or ongoing genotype acquisition rather than impaired biological clearance. Prospective cohort studies incorporating more frequent sampling, viral sequencing, quantitative HPV viral load, HIV-related immunological markers, detailed behavioral data, histologic confirmation, and non-vaccine-preventable HPV genotypes are needed to better elucidate the natural history and clinical significance of concurrent anal HPV infections.

## Figures and Tables

**Table 1:** Demographic and clinical characteristics of participants with single and multiple vaccine-preventable HPV genotype infections at baseline (T0).

Characteristic	Single Infection (*n* = 70)	Multiple Infection (*n* = 75)	*p*
Age, median (IQR)	46 (37–53)	48 (37–56)	0.283
Assigned sex at birth, *n* (%)			
Male	65 (92.9%)	73 (97.3%)	0.209
HIV status, *n* (%)			
PLWH	62 (88.6%)	66 (88.0%)	0.915
HPV vaccination, *n* (%)			
Vaccinated *	21 (30.0%)	17 (22.7%)	0.316
Baseline cytology, *n* (%)			
NEG	38 (54.3%)	23 (30.7%)	0.004
ASC	12 (17.1%)	16 (21.3%)	0.523
LSIL	20 (28.6%)	27 (36.0%)	0.340
HSIL	0 (0.0%)	9 (12.0%)	0.003

* vaccination with at least one dose of nonavalent HPV vaccine.

**Table 2:** HPV genotypes distribution in single and in multiple infection groups at baseline (T0).

HPV Type	Single Infection (*n* = 70)	Multiple Infection (*n* = 75)	*p*
HPV16	19 (27.1%)	39 (52.0%)	0.002
HPV18	4 (5.7%)	22 (29.3%)	<0.001
HPV31	7 (10.0%)	21 (28.0%)	0.007
HPV33	7 (10.0%)	20 (26.7%)	0.011
HPV45	5 (7.1%)	10 (13.3%)	0.280
HPV52	1 (1.4%)	17 (22.7%)	<0.001
HPV58	12 (17.1%)	35 (46.7%)	<0.001
HPV6	10 (14.3%)	23 (30.7%)	0.028
HPV11	5 (7.1%)	10 (13.3%)	0.280

**Table 3:** Genotype-specific repeated detection and clearance of vaccine-preventable HPV types at 12-month follow-up (T1), by baseline (T0) infection pattern (single vs. multiple infection).

HPV Type	Overall Repeated Detection	Single Infection	Multiple Infection	*p*
HPV16	44/58 (75.9%)	14/19 (73.7%)	30/39 (76.9%)	1
HPV18	15/26 (57.7%)	1/4 (25.0%)	14/22 (63.6%)	0.279
HPV31	20/28 (71.4%)	5/7 (71.4%)	15/21 (71.4%)	1
HPV33	15/27 (55.6%)	3/7 (42.9%)	12/20 (60.0%)	0.662
HPV45	9/15 (60.0%)	2/5 (40.0%)	7/10 (70.0%)	0.329
HPV52	11/18 (61.1%)	1/1 (100%)	10/17 (58.8%)	1
HPV58	34/47 (72.3%)	12/12 (100%)	22/35 (62.9%)	0.021
HPV6	19/33 (57.6%)	6/10 (60.0%)	13/23 (56.5%)	1
HPV11	6/15 (40.0%)	2/5 (40.0%)	4/10 (40.0%)	1
HPV Type	Overall Clearance			
HPV16	14/58 (24.1%)	5/19 (26.3%)	9/39 (23.1%)	1
HPV18	11/26 (42.3%)	3/4 (75.0%)	8/22 (36.4%)	0.279
HPV31	8/28 (28.6%)	2/7 (28.6%)	6/21 (28.6%)	1
HPV33	12/27 (44.4%)	4/7 (57.1%)	8/20 (40.0%)	0.662
HPV45	6/15 (40.0%)	3/5 (60.0%)	3/10 (30.0%)	0.329
HPV52	7/18 (38.9%)	0/1 (0.0%)	7/17 (41.2%)	1
HPV58	13/47 (27.7%)	0/12 (0.0%)	13/35 (37.1%)	0.021
HPV6	14/33 (42.4%)	4/10 (40.0%)	10/23 (43.5%)	1
HPV11	9/15 (60.0%)	3/5 (60.0%)	6/10 (60.0%)	1

**Table 4:** Baseline (T0) characteristics of individuals according to vaccination status, including age, assigned sex at birth, HIV status, cytology.

Characteristic	Vaccinated (*n* = 38) *	Unvaccinated (*n* = 107)	*p*
Age, median (IQR)	40 (35–47)	50 (39–56)	<0.001
Assigned sex at birth, *n* (%)			
Male	35 (92.1%)	103 (96.3%)	0.305
HIV status, *n* (%)			
PLWH	34 (89.5%)	94 (87.8%)	0.789
Baseline cytology, *n* (%)			
NEG	21 (55.3%)	40 (37.4%)	0.055
ASC	9 (23.7%)	19 (17.8%)	0.427
LSIL	5 (13.1%)	42 (39.2%)	0.004
HSIL	3 (7.9%)	6 (5.6%)	0.698

* vaccination with at least one dose of nonavalent HPV vaccine.

**Table 5:** Distribution of vaccine-preventable HPV genotypes at baseline (T0), according to HPV vaccination status.

HPV Type	Vaccinated (*n* = 38) *	Unvaccinated (*n* = 107)	*p*
HPV16	10 (26.3%)	48 (44.9%)	0.045
HPV18	8 (21.0%)	18 (16.8%)	0.559
HPV31	9 (23.7%)	19 (17.7%)	0.427
HPV33	8 (21.0%)	19 (17.8%)	0.654
HPV45	5 (13.2%)	10 (9.3%)	0.507
HPV52	2 (5.3%)	16 (14.9%)	0.157
HPV58	15 (39.5%)	32 (29.9%)	0.279
HPV6	7 (18.4%)	26 (24.3%)	0.458
HPV11	3 (7.9%)	12 (11.2%)	0.760

* vaccination with at least one dose of nonavalent HPV vaccine.

## Data Availability

The data that support the findings of this study will be made available upon reasonable request.

## Abbreviations

The following abbreviations are used in this manuscript:

HPV	Human Papillomavirus
HIV	Human immunodeficiency virus
PLWH	People living with HIV
MSM	Men who have sex with men
9vHPV	nonavalent HPV vaccine
DNA	Deoxyribonucleic acid
T0	Baseline HPV DNA testing
T1	Follow-up visit
ASC	Atypical squamous cells
LSIL	Low-grade squamous intraepithelial lesion
HSIL	High-grade squamous intraepithelial lesion
NEG	Negative

## Appendix A

**Table A1:** Exploratory multivariable logistic regression model for abnormal baseline cytology.

Outcome	Predictor	Adjusted OR (95% CI)	*p*
Abnormal T0 cytology	Multiple vs. single infection	2.65 (1.33–5.28)	0.006
	Age	0.99 (0.96–1.02)	0.482
	HPV vaccination status	0.46 (0.20–1.05)	0.065
	HIV status	1.03 (0.35–3.00)	0.965

CI = Confidence Interval; HIV = human immunodeficiency virus; OR = Odds Ratio; T0 = baseline. Outcome events: 84 abnormal baseline cytology cases. Abnormal T0 cytology was defined as ASC, LSIL, or HSIL versus negative cytology. The model was adjusted for infection pattern, age, HPV vaccination status, and HIV status. This model was considered exploratory. Multivariable models for complete HPV negativity, clearance of all baseline vaccine-preventable genotypes, cytologic regression, cytologic progression, and genotype-specific repeated detection were not performed because of limited event numbers, small genotype-specific denominators, and the risk of overfitting.
